# Treatment of Refractory Idiopathic Ischemic Priapism: A Case Report and Literature Review

**DOI:** 10.7759/cureus.63066

**Published:** 2024-06-24

**Authors:** Priyanka Desai, Jugroop Khangura, Kristy-Lyn Mcintosh, Ihab Aziz

**Affiliations:** 1 Family Medicine, Mount Sinai Hospital, Chicago, USA

**Keywords:** low-flow priapism, high-flow priapism, idiopathic priapism, erectile dysfunction, erection, recurrent priapism, nonischemic priapism, ischemic priapism, priapism

## Abstract

Priapism is a medical condition characterized by a prolonged period of penile rigidity in the absence of external sexual stimulation. Three broad categories exist for this condition: ischemic (low venous flow), nonischemic (high arterial flow), and recurrent (stuttering). Ischemic priapism is a urological emergency necessitating immediate medical attention. This literature aims to highlight the importance of prompt workup and treatment of ischemic priapism in order to prevent irreversible damage to the penis, such as erectile dysfunction and impotence. This case report presents a 35-year-old patient who developed refractory ischemic priapism in the absence of an underlying causative agent. Fortunately, through pharmacological and surgical interventions, the patient was successfully treated with complete resolution of his symptoms.

## Introduction

Priapism refers to the state in which the penis remains erect and rigid for a prolonged period of time in the absence of sexual stimulation. Although priapism can occur in all age demographics, it is most commonly seen as a bimodal distribution occurring in children between 5 and 10 years and in adults between 20 and 50 years [1].

The pathophysiology behind a penile erection involves relaxation of the cavernosal artery, precipitating increased arterial blood flow into the corpus cavernosum smooth muscle and decreased venous outflow. When the pressure within the corpus cavernosum equilibrates with the mean arterial pressure, the inflow of arterial blood ceases. When the penis reaches a rigid, erect state, it is known as an erection. A penile erection lasting longer than four hours is defined as priapism [2,3].

Priapism can be broadly categorized by its etiologies into three main types: ischemic (low venous flow), nonischemic (high arterial flow), and recurrent (stuttering).

Ischemic priapism is not only the most common form of priapism but also the most worrisome and is considered a urologic emergency. This is because impaired relaxation of the cavernosal smooth muscle causes poor outflow of deoxygenated venous blood. This can lead to painful hypoxia and acidosis of the peripheral cavernous tissue, with the potential to cause irreversible muscle damage [4]. Common causes of ischemic priapism include, but are not limited to, vasoactive medications (Papaverine, Phentolamine, Alprostadil), antipsychotic drugs (Chlorpromazine, Phenothiazine, Clozapine), antidepressants (Trazodone), illicit drugs (cocaine), hemoglobinopathies (thalassemia, sickle cell disease), neoplastic processes (melanoma, prostate cancer, renal cancer, bladder cancer), hematological diseases (leukemia), and phosphodiesterase type 5 inhibitors (Sildenafil, Tadalafil) [5-8].

Unlike ischemic priapism, nonischemic priapism does not typically require emergent medical attention as the blood is arterial and well-oxygenated in nature. Nonischemic priapism commonly occurs due to congenital arterial malformations or iatrogenic injury caused by surgical interventions or trauma to the perineum, resulting in the formation of an arterio-cavernosal fistula. The introduction of this irregular vascular connection allows for increased arterial blood flow into the cavernosal sinusoids, resulting in a high-flow vascular system.

Recurrent priapism, also known as stuttering priapism, is defined as repetitive episodes of prolonged erections, typically ischemic in nature. Recurrent priapism is an uncommon condition, and the mechanisms of action responsible for its occurrence are still not well understood [9]. Despite limited knowledge of the pathophysiology underlying the condition, some documented cases have been associated with sickle cell disease and, on rare occasions, cannabis use [10,11].

The incidence of priapism in the United States is estimated to be 0.34-5.34 per 100,000 male subjects per year [12]. In a review of 230 cases of priapism, more than 33% of cases were attributed to idiopathic causes, 21% were reactions to drugs and alcoholism, 12% were caused by trauma, 11% were caused by sickle cell anemia, and less than 1% were due to neoplasms [13].

Despite the high incidence of spontaneous idiopathic priapism, the condition remains poorly understood. This is especially concerning as a critical aspect of tailoring a treatment regimen for patients with priapism is understanding and evaluating the underlying etiology that caused the priapism. Without this critical piece of information, it is difficult to gauge future prognosis or prevent relapses of the condition.

## Case presentation

A 35-year-old male with no significant past medical history presented to the emergency department with a painful erection for the past eight hours. The patient's family history was positive for arrhythmias of unknown type in his mother and sickle cell trait in his father.

The patient reported that around 2 a.m. on the day of admission, he awoke with what he thought was a morning erection. Throughout the morning, his erection became increasingly engorged and painful. The patient attempted to relieve his symptoms with a hot shower and multiple aspirins; however, symptoms persisted.

The patient denied any trauma, sexual intercourse, or use of vasoactive medications such as sildenafil (Viagra) or tadalafil (Cialis) in the past 24 hours. The patient reported no history of similar episodes and denied any genitourinary symptoms such as dysuria, changes in urinary frequency, urinary urgency, hematuria, scrotal swelling, or testicular pain. The patient also denied gastrointestinal symptoms such as abdominal pain, diarrhea, constipation, or any constitutional symptoms such as fever, chills, malaise, and myalgia.

Upon presentation to the emergency department, the patient's vitals were significant for an elevated blood pressure of 205/116 and a heart rate of 130 beats per minute, with all other vitals within normal limits. The complete blood count (CBC) was suggestive of leukocytosis, reflected by an elevated white blood cell count of 17.81/mcL, elevated absolute neutrophils of 14.5/mcL, and elevated monocytes of 1.30/mcL. The urine drug screen, urinalysis, and complete metabolic panel (CMP) were nonsignificant. Urology was consulted, and bedside needle aspiration of the corpus cavernosum was performed successfully with 12 cc of blood withdrawn, providing temporary relief of pain.

**Figure 1:**
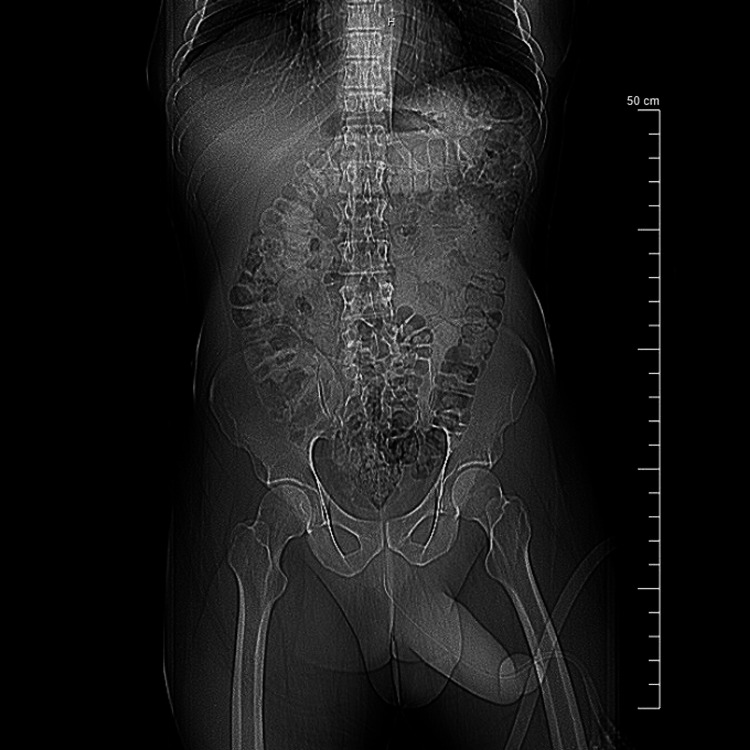
The CT scan of the abdomen and pelvis with contrast in the coronal view is unremarkable for malignancy or intra-abdominal masses. No large fluid collections are observed to suggest an abscess.

In addition, hematology was also consulted to determine if there were any underlying hemoglobinopathies or malignancies to consider. A peripheral blood smear and hemoglobin electrophoresis were both unremarkable for sickle cell disease. A coagulation panel to assess for potential veno-occlusive causes was also within normal limits. The hemolysis workup (lactate dehydrogenase and reticulocyte count) was unremarkable. Computed tomography (CT) of the abdomen and pelvis was unremarkable for malignancy or intra-abdominal/pelvic masses (Figure *1*). Based on the negative workup for underlying causes, the patient's condition was deemed to be idiopathic in nature.

The initial arterial blood gas (ABG) analysis of the aspirated blood revealed acidosis with an arterial pH of 7.0, partial pressure of arterial carbon dioxide (pCO_2_) of 60 mmHg, partial pressure of arterial oxygen (pO_2_) of 39 mmHg (reference values for typical blood gas reference values are described in Table *1*). Given the results of the ABG analysis, the patient was diagnosed with acute ischemic low-flow priapism.

**Table 1:** Typical blood gas reference values per American Urological Association guidelines in comparison to the current patient. pH: potential of hydrogen, pO_2_: partial pressure of oxygen, pCO_2_: partial pressure of carbon dioxide

Source	pH	pO_2_ (mmHg)	pCO_2_ (mmHg)
Normal arterial blood (room air)	7.4	>90	<40
Normal mixed venous blood (room air)	7.35	40	50
Acute ischemic priapism (cavernous blood)	<7.25	<30	>60
Current patient’s cavernous blood	7.0	39	60

Despite supportive pain management with ice packs and oral analgesics, the patient continued to report significant pain symptomatology with worsening penile swelling and new-onset inability to urinate. The patient was subsequently started on a tiered pharmacological pain regimen of 500 mg Tylenol, two capsules every four hours as needed for mild pain, 5/325 mg Norco (hydrocodone/ acetaminophen) one tab every six hours as needed for moderate to severe pain, and 0.5 mg intravenous Dilaudid every two hours as needed for breakthrough pain.

As per urology recommendations, other conservative measures were attempted prior to the discussion of surgical intervention. The patient underwent a subsequent penile irrigation with injectable sterile saline and phenylephrine. Despite drainage of only a limited amount of blood, pain symptoms improved significantly, and the patient was able to urinate 500 cc of urine immediately following the procedure.

Despite both interventions, the patient's blood pressure remained elevated, with a baseline of 194/124 in the left arm and 188/112 in the right arm. This elevation in blood pressure is likely secondary to the pain experienced by the patient during evaluation and workup. The patient was started on labetalol 10 mg as needed for systolic blood pressure over 180 mmHg and diastolic blood pressure over 110 mmHg.

Although dual drainage procedures resulted in temporary flaccidity of the patient's erection, the penis once again took on its erect state within 24 hours post-irrigation. After consultation with the urology team, the decision for urgent surgical management with the placement of a Winter’s shunt (corpora-cavernosal shunt) was made.

The bilateral distal corporotomy (Winter’s shunt) surgery consisted of separating the fibrotic corporal tissue from the inner lining of the tunica albuginea connective tissue. This allowed for the successful removal of fibrotic intracorporal tissue from the penis. Manual compression of the penis after removal of the fibrotic tissue expressed abundant dark blood with complete detumescence of the penis.

Despite successful surgical mediation, the patient's penis once again returned to its original painful erect state within 24 hours post-operation. Due to the patient's poor response to conservative management and given the high risk of permanent erectile dysfunction due to the prolonged duration of non-oxygenated tissue in the corpus cavernosum smooth muscle, the urology team suggested four potential options: intervention with corporal irrigation, repeat shunting, surgical decompression, or penile prosthesis. After discussing management options and evaluating the risk associated with each procedure, the patient endorsed that he wished to undergo penile surgical decompression.

During the surgery, the proximal and distal ends of the lateral left corpora were dilated, resulting in an immediate return of bright red oxygenated blood. Surgical decompression resulted in considerable detumescence of the penis.

The patient was seen post-op and endorsed a significant reduction in pain symptoms, thus allowing him to be successfully weaned off intravenous analgesics. Surgical decompression of subcutaneous edema and omission of his pain symptoms also enabled him to void approximately 300 cc of urine without difficulty.

The patient was discharged with Trimethoprim-sulfamethoxazole (Bactrim) 160/800 mg double-strength antibiotic with one tablet twice a day dosage for seven days as prophylaxis. The patient was advised to follow up with urology at an outpatient clinic following discharge. Of note, all labs, including CBC, CMP, and urinalysis, were within normal limits prior to discharge.

## Discussion

Although the diagnostic differentiation between ischemic and nonischemic priapism may be suspected based on presenting history and physical examination, confirmation must be obtained with cavernosal blood gas analysis or color duplex ultrasonography of the penis. Ischemic priapism will yield dark, viscous, hypercapnic acidic blood on blood gas analysis, while color duplex ultrasound of the perineum will reflect poor arterial flow in the cavernosal arteries due to the presence of non-oxygenated blood. In contrast, nonischemic priapism will aspirate bright red blood with normal arterial values, and Doppler ultrasound will show high arterial influx with adequate outflow due to the presence of oxygenated blood.

When managing a patient with priapism, the underlying etiology of the priapism is critical in order to accurately diagnose and develop an appropriate treatment plan.

Given the need for emergent medical attention involved with ischemic priapism, physicians should be aware of the acute workup and associated complications involved with delays in medical management. When ischemic priapism lasts longer than six hours, microscopic changes inside the penis, such as sporadic endothelial defects, may be visualized. Twelve hours after the onset of priapism, permanent structural changes occur, such as trabecular interstitial edema of the smooth muscle tissue [14]. If the priapism lasts longer than 24 hours, irreversible damage begins, and up to 90% of men cannot have normal sexual intercourse afterward. In extreme cases, when priapism lasts over 24-48 hours, permanent damage may occur, such as gangrene, resulting in partial or complete necrosis of the cavernosal smooth muscle and endothelial cells with fibroblast proliferation and fibrosis of the corpus cavernosa [15]. According to the American Urological Association guidelines, the first-line treatment for ischemic priapism includes intracorporal aspiration and a 0.5-1 mL injection of 100-500 mcg/mL concentration of a diluted alpha-adrenergic saline agent such as phenylephrine every five minutes for a total of up to three injections [2].

When medical therapy exceeds 1,000 mcg of injected diluted phenylephrine or priapism persists for over 15.5 hours, surgical intervention with a shunt may be considered to decrease corporal pressures, aid drainage, and reduce penile pain [16]. Shunting involves creating a fistula between the corpus carvernosum and corpus spongiosum to irrigate blood out of the corpora until the color changes to a bright red, indicating adequate oxygenation. Several shunting techniques, such as Winter, Ebbehoj, Al-Ghorab, and T-shaped shunts, have been documented to have successful results. Another surgical intervention is penoscrotal decompression, which involves opening and isolating the corpora to evacuate possible debris or corporal thrombi. In cases of extremely prolonged priapism causing necrosis, a shunting procedure is likely to fail, and early implantation of a penile prosthesis is recommended to prevent fibrosis [17].

Unlike ischemic priapism, non-ischemic priapism does not require emergency medical care as the blood is well-oxygenated, and spontaneous resolution has been reported in 62% of cases when left untreated. The treatment of choice for high-flow priapism, however, is embolization of the affected cavernosal artery with gel foam or an autologous blood clot to permit closure of the arterio-cavernosal fistula with rechanneling of the embolized artery. This method has been reported to restore sexual function in approximately 86% of cases [18].

## Conclusions

Spontaneous ischemic priapism is a common yet serious medical condition. The pathophysiology underlying the condition allows for irreversible damage and is therefore considered a medical emergency that requires immediate medical attention. Due to its grave ability to cause long-term repercussions, clinicians place a significant focus on the etiology underlying the condition in order to determine an effective and appropriate treatment regimen.

Despite several well-documented causes of ischemic priapism, idiopathic causes still account for the largest majority of ischemic cases. The lack of knowledge about the source of this anomaly not only presents a gap in current medical practice and poses uncertainty in medical management but, more importantly, places patients at an increased risk of recurrence in the future. This is especially concerning due to the high incidence of idiopathic priapism. For this reason, further literature outlining various cases of idiopathic priapism, including historical features, presenting symptoms, and events preceding the onset of symptoms, is warranted in order to help identify the pathophysiology underlying this condition in hopes of curtailing future occurrences and decreasing the incidence of this medical anomaly.
